# Antiseizure Effects of Fully Characterized Non-Psychoactive *Cannabis sativa* L. Extracts in the Repeated 6-Hz Corneal Stimulation Test

**DOI:** 10.3390/ph14121259

**Published:** 2021-12-03

**Authors:** Anna-Maria Costa, Lara Senn, Lisa Anceschi, Virginia Brighenti, Federica Pellati, Giuseppe Biagini

**Affiliations:** 1 Department of Biomedical, Metabolic, and Neural Sciences, University of Modena and Reggio Emilia, Via G. Campi 287, 41125 Modena, Italy; annamaria.costa@unimore.it (A.-M.C.); lara.senn@unimore.it (L.S.); 2 PhD School of Clinical and Experimental Medicine (CEM), University of Modena and Reggio Emilia, Via G. Campi 287, 41125 Modena, Italy; lisa.anceschi@unimore.it; 3 Department of Life Sciences, University of Modena and Reggio Emilia, Via G. Campi 103, 41125 Modena, Italy; virginia.brighenti@unimore.it

**Keywords:** *Cannabis sativa* L., hemp, cannabidiol, terpenes, epilepsy, 6-Hz corneal stimulation, antiseizure activity, power band spectrum, FosB/ΔFosB

## Abstract

Compounds present in *Cannabis sativa* L. preparations have recently attracted much attention in the treatment of drug-resistant epilepsy. Here, we screened two olive oil extracts from a non-psychoactive *C. sativa* variety, fully characterized by high-performance liquid chromatography and gas chromatography. Particularly, hemp oils with different concentrations of terpenes were administered at the same dose of cannabidiol (25 mg/kg/day orally), 1 h before the 6-Hz corneal stimulation test (44 mA). Mice were stimulated once a day for 5 days and evaluated by video-electrocorticographic recordings and behavioral analysis. Neuronal activation was assessed by FosB/ΔFosB immunoreactivity. Both oils significantly reduced the percentage of mice experiencing convulsive seizures in comparison to olive oil-treated mice (*p* < 0.050; Fisher’s exact test), but only the oil enriched with terpenes (K2) significantly accelerated full recovery from the seizure. These effects occurred in the presence of reduced power of delta rhythm, and, instead, increased power of theta rhythm, along with a lower FosB/ΔFosB expression in the subiculum (*p* < 0.050; Duncan’s method). The overall findings suggest that both cannabinoids and terpenes in oil extracts should be considered as potential therapeutic agents against epileptic seizures and epilepsy.

## 1. Introduction

Epilepsy is characterized by the recurrence of spontaneous seizures, and represents one of the most common neurological diseases [1]. Notably, its prevalence rate of 0.5% to 1.0% within the global population ranks epilepsy in fifth place within neurological diseases, after stroke, migraine, dementia, and meningitis [2,3]. Moreover, epilepsy is accompanied by a remarkable psychological, social, and economic burden for patients and their caregivers, which complicates the scenario of this disease [4]. Despite the efforts made to develop new anti-seizure medications (ASMs) for the treatment of epilepsy, for which several clinical trials have been performed, one third of patients still display drug-refractory epilepsy [5].

In this context, the beneficial effects of the *Cannabis sativa* L. plant in the treatment of seizures have been known since ancient times. The active ingredients of this plant are mainly ∆^9^-tetrahydrocannabinol (∆^9^-THC) and cannabidiol (CBD), the latter considered much safer and more effective in treating seizures, because it is devoid of the adverse psychotropic effects which represent the main limitation of ∆^9^-THC. Although the therapeutic potential of CBD in seizure disorders has been known for many years, it is only in the last decade that major progress has been made in characterizing its preclinical and clinical properties as a new ASM [6]. While the precise mechanism of action of CBD in humans remains unknown, there are several plausible targets engaged by CBD. The preclinical evidence strongly implicates three main molecular targets in CBD anticonvulsive properties [7]. In particular, CBD reduces neuronal excitability through functional antagonism of GPR55 receptors, desensitization of TRPV1 receptors, and inhibition of adenosine transport [7], even if other receptors and channels possibly involved in its activity cannot be excluded [8]. A pharmaceutical-grade formulation of purified CBD, derived from *C. sativa*, was evaluated in several randomized placebo-controlled adjunctive-therapy trials, which resulted in its regulatory approval for the treatment of seizures associated with Dravet syndrome, Lennox–Gastaut syndrome, and tuberous sclerosis complex [6]. Interpretation of the results of these trials was complicated by the occurrence of an interaction with clobazam in CBD-treated patients [6].

In a very recent study, cannabigerolic acid (CBGA), which is the biosynthetic precursor of all cannabinoids, was found to be more potent than CBD in reducing seizures triggered by a febrile event in a mouse model of Dravet syndrome [9]. Higher doses of CBGA also had pro-convulsant effects on other seizure types, highlighting a limitation of this cannabis constituent in comparison with CBD [9].

Cannabidiolic acid (CBDA), the direct precursor of CBD which is spontaneously generated under the action of heat and light, showed anticonvulsant activity in an acute rat model of seizures [10]. However, the chemical instability of CBDA poses challenges for its potential clinical utility [10]. All these issues would need to be overcome before another cannabinoid supersedes CBD as a new anticonvulsant drug.

A meta-analysis paper described the results of observational clinical studies on the treatment of refractory epilepsy with CBD-based products. CBD-rich extracts from *C. sativa* seem to present a better therapeutic profile than purified CBD, at least in this population of patients with refractory epilepsy [11]. This difference is probably due to synergistic effects of CBD with other compounds, in particular with terpenes, in the so-called entourage effect, but this remains to be confirmed [11]. The use of hemp extracts could offer advantages in comparison with pure CBD, in view of the above-mentioned entourage effect, due to volatile compounds, as described for both mood disorders and pain [12,13]. The bottleneck of this approach is related to the required standardization of the plant material, as well as the extraction method, in order to ensure not only the efficacy and the reproducibility of the pharmacological action, but also its safety. Indeed, it is well documented that there is a high degree of variability in the composition of medical cannabis oils on the basis of the extraction method [14].

To further evaluate the potential use of non-psychotropic *C. sativa* extracts that are highly rich in CBD as a new therapeutic treatment for resistant epilepsy, we tested CBD and *C. sativa* extracts in the 6-Hz corneal stimulation mouse model [15,16,17,18,19]. Specifically, we aimed to test the in vivo antiseizure effect of olive oil extracts from hemp inflorescences, which were fully characterized for their content of both cannabinoids and terpenes. Even though it was challenging to study the chemical composition of plant extracts, in view of their complex chemical composition, the full characterization of both cannabinoids and terpenes was made possible and reliable thanks to specific and fully validated analytical methods based on high-performance liquid chromatography (HPLC) and gas chromatography (GC) [20,21,22,23,24]. The influence of the compounds found in the extracts, specifically terpenes in combination with cannabinoids, on the experimental results obtained after the administration of the oil extracts and pure CBD in mice is illustrated in detail.

## 2. Results

### 2.1. Qualitative and Quantitative Analysis of Cannabinoids in Hemp Oils

The identification of the secondary metabolites in hemp oils was carried out by ultra-high-performance liquid chromatography (UHPLC), coupled with high-resolution mass spectrometry (HRMS). In order to verify the presence of additional cannabinoids in hemp, for which analytical standards were not available, a target metabolomic analysis was employed. This was performed using a compiled list of 213 cannabinoids, on the basis of those described in the literature [25], and additional potential cannabinoids that were added according to the biosynthesis and decomposition pathways previously described [25,26,27].

The MS and MS/MS data for the compound identified in hemp oils are shown in Table 1.

Cannabinoic acids showed a higher intensity of signals in the negative ion mode, as opposed to the neutral compounds which ionized better in the positive ion mode. A total of 15 neutral cannabinoids were identified in the positive ion mode, while 6 cannabinoic acids were identified in the negative ion mode. Cannflavins A and B, which are isoprenic flavones present in minor amounts in hemp, were detected in the positive ion mode [21]. It should be pointed out that the qualitative profile was the same for the two hemp oils, due to the application of the same extraction procedure for this class of compounds.

**Figure 1:**
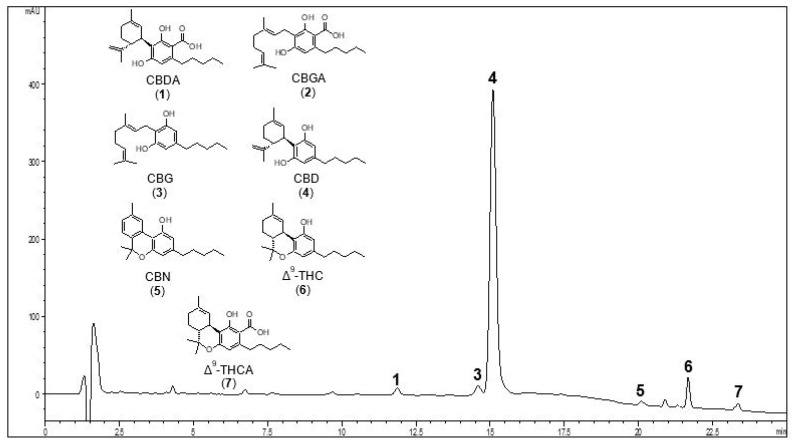
Representative HPLC-UV chromatogram of hemp oil 2 (K2) recorded at 210 nm. The peaks of the main neutral and acid cannabinoids are shown. For peak identification, see Table 2. The chemical structures of the compounds selected for quantitative analysis are shown in the slot on the left.

Among all the identified cannabinoids, the most representative ones were further quantified by HPLC-UV. Figure 1 shows a representative HPLC-UV chromatogram of K2 hemp oil, acquired at 210 nm. The most abundant peak is that of CBD, at a retention time of 15.4 min.

Table 2 shows the quantitative results obtained from the HPLC-UV analysis of cannabinoids both in K1 and K2 oils. The data revealed that the decarboxylation process had successfully occurred for both the oils. As a matter of fact, ∆^9^-THCA, CBDA, and CBGA were under the limit of quantification (LOQ) in both cases. The content of neutral cannabinoids in K1 and K2 oils was of the same order of magnitude.

### 2.2. GC-MS Analysis of Terpenes in Olive Oil Extracts

Hemp volatile compounds in the oils were analyzed by GC-MS and identified according to their MS spectra, which were compared with available data in the MS spectral library.

**Figure 2:**
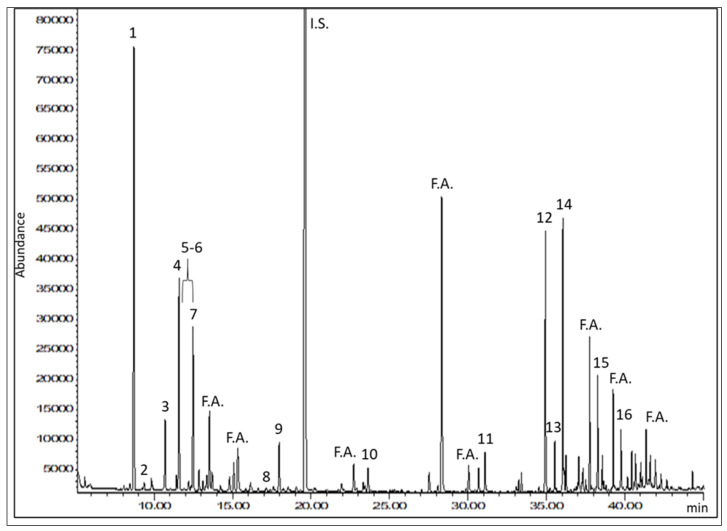
Representative GC-MS selected ion monitoring (SIM) chromatogram of hemp oil 2 (K2), obtained by monitoring the ion at 93 *m*/*z*. It is possible to see the peak of the internal standard (I.S.). Fatty acids (F.A.) from olive oil, used as the extraction solvent, eluted among the terpenes of interest. For peak identification, see Table 3.

As shown in Figure 2 and Table 3, the GC analysis allowed us to identify 16 compounds in samples K1 and K2.

The two samples displayed the same qualitative profile, since these compounds were identified in both oils. Peaks from 1 to 11 in Figure 2 were identified as monoterpenes, while peaks eluted later consisted of sesquiterpenes and caryophyllene oxide, which is a sesquiterpenoid. Among the components identified, the most representative terpenes were α-pinene and β-caryophyllene. The terpenes found in these hemp oils were the same as those previously described for dried hemp inflorescences and essential oil [21,22].

As to the quantitative analysis, the most representative terpenes in the oils, on the basis of the full-scan GC-MS data, were quantified in the SIM mode, using calibration curves built with an appropriate I.S. Quantitative data for samples K1 and K2 are shown in Table 4.

The terpene content of sample K1 was found to be very low, having only α-pinene above the LOQ. On the other hand, K2 showed consistently higher terpene concentrations. In particular, α-pinene represented the most abundant compound among monoterpenes in K2 oil, with a concentration of 11.65 µg/mL, followed by limonene, β-myrcene, and linalool. As for the sesquiterpenes, β-caryophyllene was the most abundant compound, with a concentration of 26.61 µg/mL in K2 oil, followed by α-humulene and caryophyllene oxide.

### 2.3. Characterization of the Seizures Induced by 6-Hz Corneal Stimulations

**Figure 3:**
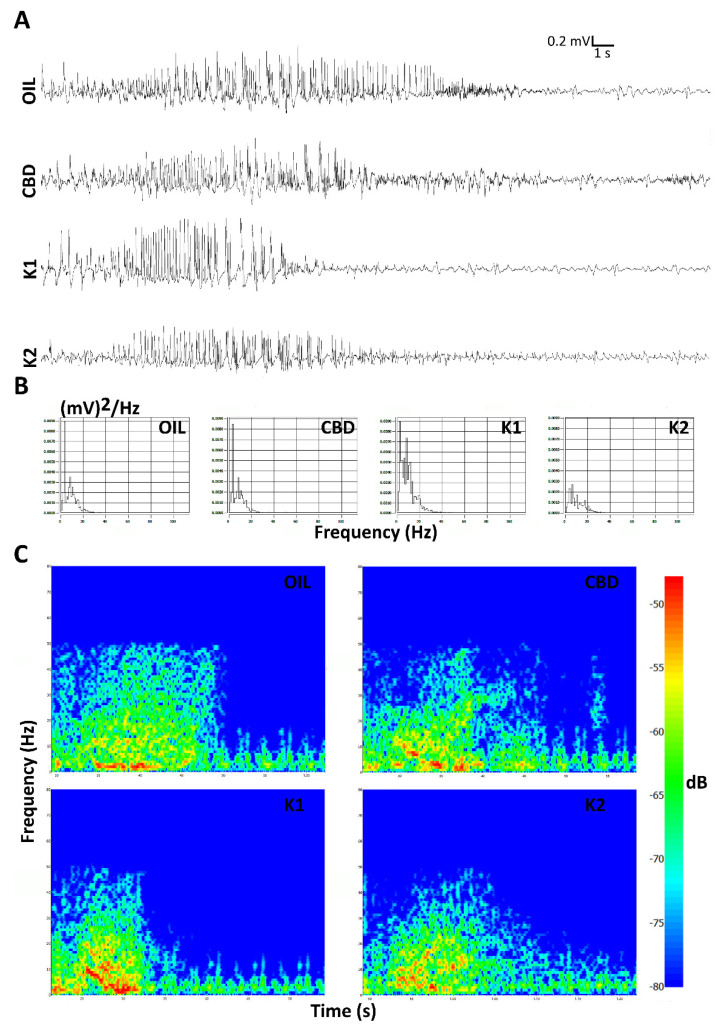
Seizures induced after the last session of 6-Hz corneal stimulation are illustrated. Seizures induced in K1- and K2-treated mice seemed to partially differ from those displayed by mice treated with either CBD alone or olive oil in the last session of the 6-Hz test. In (**A**), electrocorticographic (ECoG) traces are reported for all treatment groups. The power spectral density and power attenuation maps are represented, respectively, in (**B**,**C**). Scale: 0.2 mV/s. OIL, olive oil; CBD, cannabidiol; K1, hemp oil 1; K2, hemp oil 2.

Daily seizures were induced in mice by electroshocks delivered to both eyes for 5 consecutive sessions with intervals of 24 h. After the last session of 6-Hz corneal stimulation, seizures induced in K1- and K2-treated mice seemed to partially differ from those displayed by mice treated with a low dose of CBD (25 mg/kg) or olive oil (Figure 3A). This observation was also supported by considering the power spectral density referred to the first 10 s after the 6-Hz stimulation (Figure 3B). Moreover, the hypothesis was also in agreement with the power attenuation maps related to the electrocorticographic (ECoG) traces (Figure 3C).

### 2.4. Different Responses to Treatment

**Figure 4:**
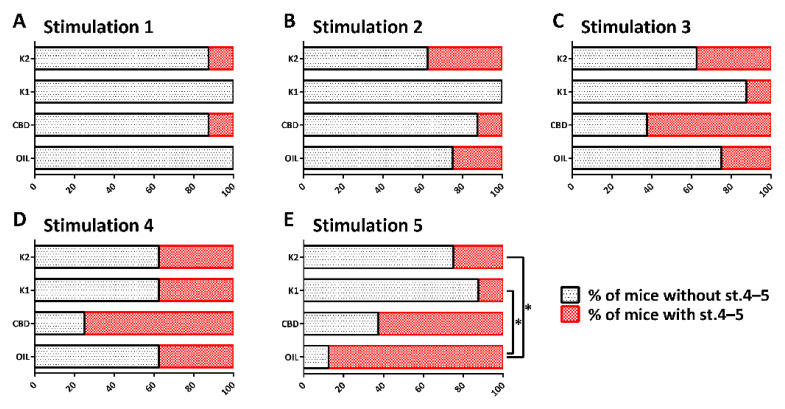
Percentage of mice developing (in red), or not developing (in black), generalized tonic–clonic convulsions, with (stage 5, st. 5) and without loss of posture (st. 4), after each session of 6-Hz corneal stimulation. The repeated 6-Hz test induced progressive changes in the seizure severity (**A**–**E**) and, at the end, the percentage of K1- and K2-treated mice developing st. 4–5 seizures was significantly reduced in comparison to olive oil-treated mice. * *p* < 0.050; Fisher’s exact test. OIL, olive oil; CBD, cannabidiol; K1, hemp oil 1; K2, hemp oil 2.

The repeated 6-Hz test induced progressive changes in the seizure severity, and percentage of mice developing generalized tonic–clonic convulsions, with and without loss of posture (Figure 4A–E). In comparison to the olive oil-treated group, the changes were not significantly different in CBD-, K1-, and K2-treated mice up to the fourth session of the 6-Hz test. After 5 sessions of 6-Hz corneal stimulation, 7 out of 8 mice treated with olive oil (87.5%) and 5 out of 8 mice treated with a low dose of CBD (62.5%) developed generalized tonic–clonic convulsions, with and without loss of posture. At variance, only 1 out of 8 mice treated with a low dose of K1 (12.5%) and 2 out of 8 mice treated with a low dose of K2 (25.0%) developed generalized tonic–clonic convulsions, with and without loss of posture. By comparing the percentage of olive oil-treated mice developing stage 4–5 seizures to those observed in the other groups, we found a significant difference in comparison to the K1- (*p* = 0.010; Fisher’s exact test) and K2-treated groups (*p* = 0.041). Instead, no differences were reported between the mice treated with olive oil and CBD, when CBD (25 mg/kg/day orally) was administered at the same dose of K1 and K2.

### 2.5. Characterization of the Duration of Ictal ECoG Recordings and Time to Recover from Seizures

**Figure 5:**
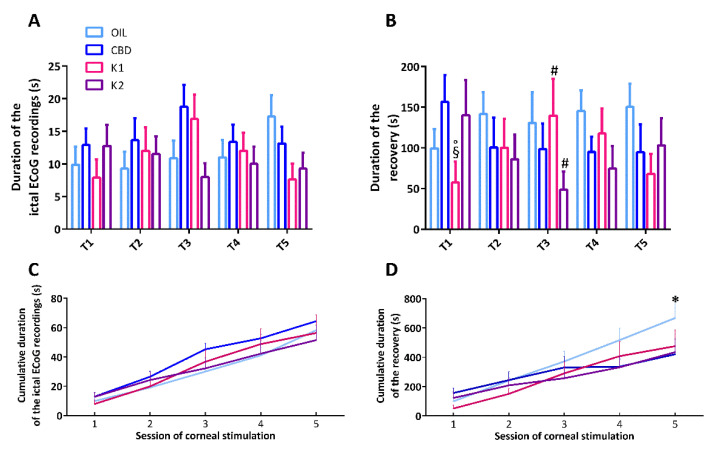
Duration of ictal electrocorticographic (ECoG) recordings, time to recover from seizures, and the cumulative effect of the treatments. All mice (*n* = 32) were treated with CBD (*n* = 8), K1 (*n* = 8), K2 (*n* = 8), or olive oil (*n* = 8). In (**A**), no treatment had beneficial effects on the total duration of seizures after the 6-Hz corneal stimulations. In (**B**), K1 and K2 had occasional beneficial effects on time to recover from seizures. In (**C**), no treatment had beneficial effects on the cumulative duration of the ictal ECoG recordings, but this cumulative duration significantly increased from the first to last session of stimulation in all groups. In (**D**), the cumulative duration of recovery significantly increased from the first to last session of stimulation in all groups, but only K2 had a significant cumulative effect on the time to recover from seizures. * *p* < 0.050 K2 vs. OIL; ^§^ *p* < 0.050 K1 vs. CBD; ° *p* < 0.050 K1 vs. K2; ^#^ *p* < 0.050 vs. session 1; Duncan’s method. T1–T5, from the first to the fifth session of corneal stimulation; OIL, olive oil; CBD, cannabidiol; K1, hemp oil 1; K2, hemp oil 2.

The duration of the ictal ECoG recordings and the time to recover from seizures were analyzed by two-way (treatment × time) repeated measures analysis of variance (ANOVA). This analysis showed no statistically significant effect of both treatment (F(3,112) = 0.832, *p* = 0.488) and time for the duration of the ictal ECoG recordings (F(4,112) = 0.723, *p* = 0.578). Similarly, there was no statistically significant interaction between treatment and time for the duration of the ictal ECoG recordings in mice (F(12,112) = 1.684, *p* = 0.080), indicating that all treatments did not modify the duration of the ictal ECoG (Figure 5A). Furthermore, the analysis showed no statistically significant effect of treatment (F(3,107) = 0.932, *p* = 0.438), nor time, for the duration of recovery from seizures (F(4,107) = 0.0877, *p* = 0.986). However, there was a statistically significant interaction between treatment and time (F(12,107) = 1.876, *p* = 0.045). Precisely, time to recover from seizures was shorter in K1-treated mice compared to CBD and K2 (*p* < 0.050; Duncan’s method), after the first session of 6-Hz corneal stimulation. K1 seemed to display a beneficial effect only in the first, compared to the third, session of stimulation (*p <* 0.050). At variance, K2 displayed beneficial effects on recovery only after 3 sessions of 6-Hz corneal stimulations (*p* < 0.050) (Figure 5B).

To evaluate the overall duration of seizures induced by the various sessions of 6-Hz corneal stimulation, we also analyzed the cumulative duration of ictal ECoG recordings and recovery by summing up the respective time intervals. A two-way repeated measures ANOVA showed no statistically significant effect of treatment (F(3,110) = 0.515, *p* = 0.676), nor an interaction between treatment and time (F(12,110) = 1.069, *p* = 0.393). As expected, a significant effect of time (F(4,110) = 122.213, *p* < 0.001) was observed for the cumulative duration of the ictal ECoG recordings (Figure 5C). Then, the analysis showed no statistically significant effect of treatment (F(3,108) = 0.738, *p* = 0.538) and a predictabable significant effect of time for the cumulative duration of recovery (F(4,108) = 84.926, *p* < 0.001). Interestingly, there was a statistically significant interaction between treatment and time for the cumulative duration of recovery (F(12,108) = 2.737, *p* = 0.003), which was reduced by K2, compared to olive oil (*p* < 0.050), after the last 6-Hz test (Figure 5D).

### 2.6. Analysis of the Power Band Spectrum

**Figure 6:**
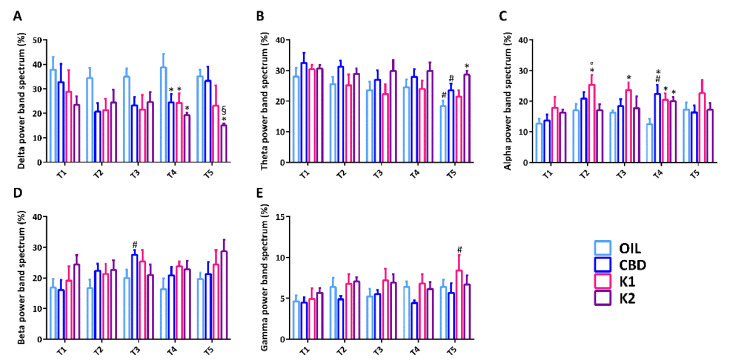
Power band spectrum analysis after each session of the 6-Hz test. All mice (*n* = 32) were treated with CBD (*n* = 8), K1 (*n* = 8), K2 (*n* = 8), or olive oil (*n* = 8). A relative indication of distribution of power over the frequency, expressed in percentage, was determined in seizures induced by the 6-Hz corneal stimulation (**A**–**E**). * *p* < 0.050 vs. OIL; ^§^ *p* < 0.050 vs. CBD; ° *p* < 0.050 vs. K2; ^#^ *p* < 0.050 vs. first session of the same group, with the same treatment; Duncan’s method. T1–T5, from the first to the fifth session of corneal stimulation; OIL, olive oil; CBD, cannabidiol; K1, hemp oil 1; K2, hemp oil 2.

The power band spectrum, expressed in percentage, was analyzed by a two-way ANOVA. In particular, the percentage of delta power showed a significant effect of treatment (F(3,108) = 8.971, *p* < 0.001), but no significant effect of time for delta power (F(4,108) = 0.748, *p* = 0.562). There were no statistically significant interactions between treatment and time for delta power in mice (F(12,108) = 0.630, *p* = 0.812). Delta power was not statistically different between groups until the third session of 6-Hz corneal stimulation, but it was significantly decreased in all groups in comparison to olive oil-treated mice in the fourth session of 6-Hz corneal stimulation (*p* < 0.050; Duncan’s method). In the last 6-Hz test, delta power remained significantly lower only in K2-treated mice in comparison to oil- and CBD-treated mice (Figure 6A).

The percentage of theta power showed a significant effect of treatment (F(3,109) = 5.381, *p* = 0.002) and time for theta power (F(4,109) = 3.996, *p* = 0.005). However, there was no statistically significant interaction between treatment and time (F(12,109) = 0.410, *p* = 0.957). In comparison to the first 6-Hz test, the percentage of theta power was significantly reduced in olive oil- and CBD-treated mice in the last session of 6-Hz stimulation (*p* < 0.050). At variance, theta power was significantly higher in the K2-treated mice in comparison to the oil-treated mice, in the same session of 6-Hz stimulation (*p* < 0.050) (Figure 6B).

Similarly, the percentage of alpha power showed a significant effect of treatment (F(3,109) = 6.781, *p* < 0.001) and time (F(4,109) = 2.473, *p* = 0.049). There was no significant interaction between treatment and time (F(12,109) = 0.934, *p* = 0.561). In comparison to the olive oil-treated mice, alpha power was significantly higher in the K1-treated mice from the second to the fourth sessions of 6-Hz stimulation (*p* < 0.050). In the second 6-Hz test, alpha power was also markedly higher in the K1-treated mice in comparison to the K2-treated mice (*p* < 0.050). Alpha power was also significantly increased in CBD- and K2-treated groups, in comparison to the olive oil-treated group, in the fourth session of 6-Hz corneal stimulation (*p* < 0.050) (Figure 6C).

The percentage of beta power showed a significant effect of treatment (F(3,108) = 3.511, *p* = 0.018) but not time (F(4,108) = 1.388, *p* = 0.243). Moreover, there was no significant interaction between treatment and time for beta power (F(12,108) = 0.663, *p* = 0.783). In comparison to the first 6-Hz test, beta power was significantly higher in the CBD-treated mice in the third session of 6-Hz corneal stimulation (*p* < 0.050) (Figure 6D).

Furthermore, the percentage of gamma power showed a significant effect of treatment (F(3,107) = 3.939, *p* = 0.010) but not time (F(4,107) = 2.137, *p* = 0.081). Also, there was no significant interaction between treatment and time for gamma power (F(12,107) = 0.368, *p* = 0.972). In comparison to the first 6-Hz test, gamma power was significantly higher in the K1-treated mice in the last session of 6-Hz corneal stimulation (*p* < 0.050) (Figure 6E).

### 2.7. FosB/ΔFosB Immunoreactivity

**Figure 7:**
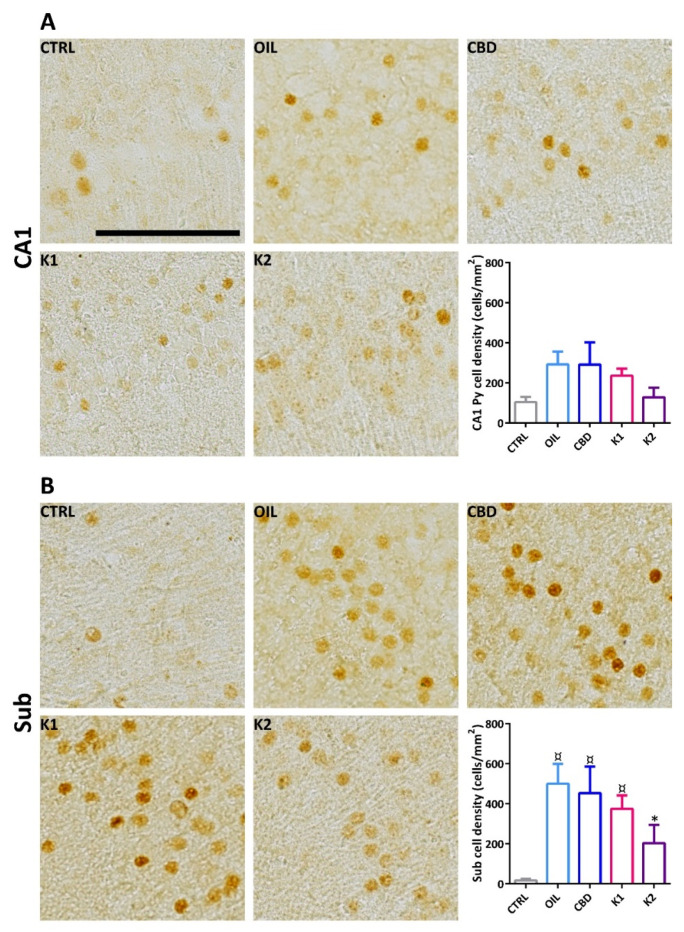
FosB/ΔFosB immunoreactivity in the hippocampal CA1 region and in the subiculum (Sub) of mice treated with CBD (*n* = 6), K1 (*n* = 6), K2 (*n* = 6), or olive oil (*n* = 6), and exposed to different sessions of 6-Hz corneal stimulation. FosB/ΔFosB immunoreactivity was also illustrated in a representative unstimulated control (Ctrl, *n* = 6). Particularly, FosB/ΔFosB immunoreactivity in the CA1 was reported in (**A**), whereas that of the Sub, in (**B**). ^¤^ *p* < 0.050 vs. CTRL; * *p* < 0.050 vs. OIL; Duncan’s method. Scale: 0.1 mm. CTRL, control; OIL, olive oil; CBD, cannabidiol; K1, hemp oil 1; K2, hemp oil 2.

We evaluated the effects of repeated exposure to 6-Hz corneal stimulation (44 mA) on FosB/ΔFosB immunoreactivity in the stratum pyramidalis of the cornu ammonis 1 (CA1) region (Figure 7A), and in the subiculum (Figure 7B). FosB/ΔFosB levels were barely detectable in all sampled regions of unstimulated control mice. In comparison to the non-stimulated control group, neuronal expression was increased in all groups. These changes were significant in the Sub (*p* < 0.050; Duncan’s method), but not in CA1. Interestingly, a prominent increase in FosB/∆FosB levels was observed in the Sub of oil-, CBD-, and K1-treated mice in comparison to the non-stimulated control group. On the other hand, treatment with K2 prevented a significant increase in FosB/∆FosB levels in the Sub.

We tested the antiseizure activity of two different hemp oils, which were compared to CBD at a concentration similar to that found in the extracts. The results of our investigation suggest that both extracts exerted an antiseizure activity, and they were more efficient than CDB when tested as the only administered anticonvulsant. Additionally, the extract enriched with terpenes was advantageous in affording a faster recovery from seizures. This effect was observed in the presence of preserved theta activity in the ECoG and restrained FosB/ΔFosB expression in the subiculum. These findings confirm the validity of hemp oils to address seizures, and further suggest a CBD-independent role of terpenes as an ASM [28,29,30].

As for the influence of the chemical composition of hemp oils on bioactivity data, the difference in the content of volatile compounds between hemp oils K1 and K2 could be responsible for the results obtained in the 6-Hz seizure test, there being neutral cannabinoids present in a very similar amount in both the samples. In particular, the quantitatively higher content of terpenes in K2 may act in a synergistic way with cannabinoids to enhance the anticonvulsant effect of K2 compared with K1 and with pure CBD. A similar entourage effect between cannabinoids and terpenes was previously described for mood disorder, as well as for pain [12,13]. Furthermore, some representative terpenes of K2, such as β-caryophyllene (a CB_2_ receptors agonist), β-myrcene and limonene have displayed anticonvulsant activity against seizures induced by pentylenetetrazol [31,32]. Moreover, linalool has been able to act as an anticonvulsant in quinolinic acid-induced seizures in vivo [33] and to alleviate maximal electroshock-induced seizures in mice [34].

## 3. Discussion

Although both K1 and K2 reduced the occurrence of convulsive seizures, K2 appeared to promote a prompt recovery from seizures, therefore suggesting a possible advantage. Interestingly, this effect was observed in the presence of better-preserved theta activity in the ECoG, which was kept at the same level in all session of 6-Hz corneal stimulation, whereas it declined in the other treatment groups. We already observed changes in the power of the theta band in other experiments, which suggested a role for this frequency in seizure characteristics [35,36]. Specifically, during status epilepticus evoked by kainic acid in rats, a drop in theta power preceded the appearance of generalized tonic–clonic seizures, the most severe epileptic activity observed in this model [35]. However, in the present experiment, theta power seemed to be more related to the faster recovery from seizures observed in the K2-treated group, in which the involvement of reduced activation of subicular neurons could also be hypothesized. The subiculum is a region of the hippocampal formation able to promote seizure activity, and it is also involved in refractoriness to some ASMs [37,38,39]. Thus, the observed effect of K2 on the subiculum in the 6-Hz model of corneal stimulation, which was suggested as a model of resistance to ASMs, encourages further studies to assess the possible efficacy of this hemp oil in addressing difficult to treat seizures.

Other effects of treatments on the power spectrum of the ECoG were also observed. Most of them were apparently not related to the anticonvulsant effects of the treatment, with the exception of delta power. In addition, this spectrum band was kept at the same level in the K2 group until the last session of 6-Hz corneal stimulation. In a previous experiment, we reported that the power of the delta band in postictal depression was significantly related to the remarkable increase in seizure occurrence found in epileptic rats after the withdrawal of levetiracetam, previously administered for a week as an ASM [36]. For this reason, the changes in the power band observed in the other treatment groups could have contributed to the seizure characteristics found in the present experiment.

It is important to highlight that the chronic administration of CBD alone might markedly induce anticonvulsant and antiepileptogenic effects [40]. However, the crucial role of the volatile component of K2 in the overall activity of the extract, together with the presence of CBD, could be supported by the fact that CBD alone, at a low dose, seemed not to be effective [41]. Indeed, CBD injected intraperitoneally at a dose of 25 mg/kg did not significantly modify the seizure thresholds in the maximal electroshock seizure threshold test and the 6-Hz-induced seizure test in mice. At variance, when injected at higher doses of 50 and 100 mg/kg, it was significantly powerful [41]. Thus, our approach, using a dose below the threshold of CBD, definitely unmasked the anticonvulsant properties of the other compounds present in hemp oils.

## 4. Materials and Methods

### 4.1. Chemical and Solvents

Stock methanolic solutions (1.0 mg/mL) of cannabidiol (CBD), cannabigerol (CBG) and cannabinol (CBN), as well as stock acetonitrile solutions (1.0 mg/mL) of cannabidiolic acid (CBDA) and cannabigerolic acid (CBGA), were purchased from Cerilliant Corporation (Round Rock, TX, USA).

Stock 2-propanol solutions (1.0 mg/mL) of α-pinene, β-pinene, β-myrcene, limonene, linalool, β-caryophyllene, α-humulene, caryophyllene oxide and *trans*-nerolidol were obtained from Agilent Technologies (Santa Clara, CA, USA).

Acetonitrile (ACN), formic acid (HCOOH), *n*-hexane, methanol (MeOH), 2-propanol and methyl-octanoate were purchased from Sigma Aldrich s.r.l. (Milan, Italy). Water (H_2_O) was purified using a Milli-Q Plus185 system from Millipore (Milford, MA, USA).

Extra virgin olive oil was of European Pharmacopoeia grade. CBD powder (>99%) and hemp female inflorescences (Kompolti variety), listed within the EU database of admitted agricultural species, were purchased from Materia Medica Processing (Bolzano, Italy).

### 4.2. Animals

All experiments were performed according to the Italian Act (DM 26/2014) and the European Directive 2010/63/EU. Thirty-eight male CD-1 mice (Charles River, Calco, Italy), with initial weights of 15–20 g, were housed in a specific pathogen-free facility with a controlled environment and ad libitum access to water and food. All efforts were made to refine procedures and protect the animals’ welfare.

### 4.3. Preparation of the Olive Oil Extracts

Hemp inflorescences were crushed before the decarboxylation step and extraction with olive oil. Decarboxylation allows for more standardized extracts, avoiding the spontaneous conversion of cannabinoic acids into their neutral counterparts during storage of the extract [14].

All hemp oils were prepared using a drug–solvent ratio of 1:10 (*w*/*v*), as previously described [14]. To prepare hemp oil 1 (K1), not preserving volatile components, 5 g of dried plant material were decarboxylated in a beaker, placed into an oven at 110 °C for 15 min, and then at 120 °C for 60 min. The plant material was extracted with 50 mL of olive oil with a turbo-emulsifier for 10 min, keeping the temperature below 40 °C. This operation was repeated three times. The third time, an additional 4 min of sonication were applied. The combined oil extracts were finally filtered to remove the solid residue.

To prepare hemp oil 2 (K2), 5 g of dried plant material were decarboxylated in an oven at 115 °C for 1.30 h in a sealed flask, in order to retain the volatile compounds inside. The plant material was then extracted with 50 mL of olive oil directly in the flask, following the same extraction procedure described for K1.

CBD oil was prepared by dissolving a weighted amount of pure compound (225 mg) in 50 mL of olive oil. Hemp oils were diluted to 1:50 and 1:5 with 2-propanol prior to the injection into the HPLC and GC systems, respectively.

### 4.4. UHPLC-HRMS Analysis

Qualitative analysis of hemp oil extracts was performed by means of UHPLC-HRMS. The analyses were performed on a Thermo Scientific (Waltham, MA, USA) UHPLC Ultimate 3000, equipped with a vacuum degasser, a binary pump, a thermostated autosampler, a thermostated column compartment, and a Q-Exactive Orbitrap mass spectrometer, with a heated electro-spray ionization (HESI) source. An Ascentis Express C_18_ column (150 mm × 3.0 mm I.D., 2.7 µm, Supelco, Bellefonte, PA, USA) was used.

With regard to olive oil hemp extracts, a ternary A/B/C multistep gradient (solvent A: 0.1% HCOOH in H_2_O, solvent B: 0.1% HCOOH in ACN, and solvent C: MeOH) was used. Solvent C was kept constant at 5% throughout the run. On the basis of previous work [25], the multistep gradient program was established as follows: 0–2 min from 50 to 67% B, which was kept for 4 min; 6–10 min from 67 to 90% B, which was kept for 4 min; and 14–15 min from 90 to 50% B, which was kept for 5 min, for re-equilibration of the system prior to the next injection. A flow rate of 0.3 mL/min was used. The column temperature was 30 °C. The injection volume was 1.0 μL.

MS acquisition was carried out with a heated electro-spray ionization (ESI) source operated both in the positive and negative ion modes. As for the MS detector, the source parameters were set as follows: sheath gas (N_2_) 40, auxiliary gas (N_2_) 30, auxiliary gas temperature 290 °C, and electrospray voltage 3.5 kV (+) and 3.2 kV (−). The analyses were acquired in the full mass data-dependent (FM-dd-MS^2^) mode at a resolving power of 35.000 full width at half maximum (FWHM). The other mass analyzer parameters were set as follows: scan range *m*/*z* 150–2000, automatic grain control (AGC) target 1 × 10^6^ ions in the Orbitrap analyzer, ion injection time 100 ms, and isolation window for the filtration of the precursor ions *m*/*z* 1.0. The fragmentation of precursors ions was performed at 20, 30, and 50 as normalized collision energies (NCE).

### 4.5. HPLC-UV Analysis

HPLC-UV analyses were performed on an Agilent Technologies (Waldbronn, Germany) modular model 1260 Infinity II system, consisting of a quaternary pump, a manual injector, and a UV variable wavelength detector. Chromatograms were recorded by using an Agilent OpenLab ChemStation (Rev. C.01.10). An Ascentis Express C_18_ column (150 mm × 3.0 mm I.D., 2.7 µm, Supelco, Bellefonte, PA, USA) was used, with a mobile phase composed of 0.1% HCOOH in both (A) H_2_O and (B) ACN. The gradient elution was as follows: 0–13 min 60% B, 13–17 min from 60 to 80% B, 17–22 min from 80 to 90% B, which was kept for 8 min. The post-running time was 15 min. The flow rate was 0.4 mL/min. The sample injection volume was 3 µL. Chromatograms were acquired at 210 nm (for decarboxylated cannabinoids) and at 220 nm (for cannabinoic acids). Calibration curves for target compounds were constructed at five calibration levels in the range of 2.5–100 µg/mL, by plotting the peak areas of the analytes vs. their concentration. ∆^9^-THC and ∆^9^-THCA were quantified on the basis of the calibration curves built for CBD and CBDA. Two injections were performed for each sample. The value corresponding to the limit of quantification (LOQ) was 2.5 µg/mL for each cannabinoid, while the limit of detection (LOD) was 0.8 µg/mL.

### 4.6. GC-MS Analysis

GC-MS analysis was carried out on a 7890B gas chromatograph coupled with a 5977B GC/MSD mass spectrometer (Agilent Technologies, Germany). Compounds were separated on an Agilent Technologies HP-5 cross-linked poly-5% diphenyl-95% dimethyl polysiloxane (30 m × 0.32 mm i.d., 0.25 μm film thickness) capillary column. The column temperature was initially set at 45 °C, then increased at a rate of 2 °C/min up to 100 °C, then raised to 250 °C at a rate of 5 °C/min, and again raised up to 280 °C at a rate of 11 °C/min, and finally held for 15 min. The injection volume was 1.0 µL, with a split ratio of 1:15. Helium was used as the carrier gas at a flow rate of 1.2 mL/min. The injector, transfer line and ion-source temperature were 250, 280 and 230 °C, respectively. Mass spectra were acquired with an electronic ionization (EI) source at 70 eV, operating in the full-scan acquisition mode in the range of 40–400 *m*/*z*. MS spectra of eluting peaks were compared with those reported in the NIST mass spectral database (version 1.4)

The identification of terpenes was performed by the comparison of the experimental linear retention index (*LRI*) values obtained for the chromatographic peaks with those reported in the literature for the same plant material [22]. In addition, a mass spectra database search for the compounds present in the chromatogram, acquired in the full-scan mode, was performed.

For quantitative analysis, the selected ion monitoring (SIM) mode was applied to monitor the most intense ion at 93 *m/z* for all the terpenes of interest, and the ion at 74 *m/z* for methyl-octanoate used as the I.S. Calibration curves for target compounds were constructed at five concentration levels in the range of 1–50 µg/mL, by plotting the peak areas of the analytes vs. their concentration. The concentration of the I.S. was 25 µg/mL. Two injections were performed for each level. The concentration value corresponding to the LOQ was 1.0 µg/mL for limonene, linalool, β-caryophyllene, *trans*-nerolidol and caryophyllene oxide, and 0.5 µg/mL for α-pinene, β-pinene, β-myrcene and α-humulene. The concentration value corresponding to the LOD was 0.3 µg/mL for limonene, linalool, β-caryophyllene, *trans*-nerolidol and caryophyllene oxide, and 0.2 µg/mL for α-pinene, β-pinene, β-myrcene and α-humulene.

### 4.7. Experimental Design

Thirty-eight mice were implanted with electrodes and randomly divided into 5 groups: (i) 6 control mice were neither treated nor stimulated, (ii) 8 mice received olive oil alone, (iii) 8 mice received CBD (25 mg/kg/day orally) dissolved in olive oil, (iv) 8 mice received K1 (25 mg/kg/day orally) dissolved in olive oil, and (v) 8 mice received K2 (25 mg/kg/day orally) dissolved in olive oil. Except for the control group, the remaining mice were stimulated once and allowed to recover for 24 h before being stimulated for up to 5 sessions.

All 6-Hz stimulated mice (*n* = 32) were considered for behavior and video-ECoG recordings. At variance, a total of 6 animals per group (*n* = 30) were considered for immunohistochemical staining. In this case, the control mice were used to determine basal levels of the investigated markers, because they were handled and exposed to the same procedure as the others.

### 4.8. Electrodes Implatation and v-ECoG Recordings

Before electrode implantation, anesthesia was induced with volatile isoflurane (4% induction and 1–2% maintenance), and deep anesthesia was assessed by deep breathing and loss of tail and eye reflexes. Accordingly, the skin was shaved, disinfected with povidone-iodine 10% (Betadine^®^ skin solution; Meda Pharma, Milano, Italy), cut, and opened to expose the skull.

Guiding holes were drilled and epidural electrodes (stainless steel Ø D 1 mm; PlasticsOne, Roanoke, VA, USA) were implanted in the frontal (bregma 0 mm, 3 mm lateral from midline) and occipital cortices (bregma −3.5 mm, 3 mm lateral from midline) of both hemispheres. One electrode was implanted below lambda on the midline in all mice and used as a reference.

All electrodes were connected through steel wire to terminal gold pins (Bilaney Consultant GmbH, Düsseldorf, Germany) inserted in a plastic pedestal (PlasticsOne) cemented on the heads. After surgery, gel containing 2.5 g lidocaine chloride, 0.5 g neomycin sulfate, and 0.025 g fluocinolone acetonide (Neuflan^®^ gel; Molteni Farmaceutici, Scandicci, FI, Italy) was applied to minimize acute pain and risk of infection. All mice were monitored until complete recovery from anesthesia and housed in single cages without grids or environmental enrichments to reduce the risk of headset loss.

The ECoG was recorded during all sections of corneal stimulation via cable connection between the headset and preamplifiers. Electrical activity was digitally filtered (0.3 Hz high-pass, 500 Hz low-pass), acquired at 1 kHz per channel, and stored on a personal computer after the mathematical subtraction of traces of recording electrodes from traces of reference electrodes, using a PowerLab8/30 amplifier connected to 4 BioAmp preamplifiers (AD Instruments; Dunedin, Otago, New Zealand). Videos were digitally captured through a camera connected to the computer and synchronized to the ECoG traces through a LabChart 8 PRO internal trigger.

### 4.9. Corneal Stimulation Protocol

Corneal stimulation was performed once a day for 5 days, and was preceded by the application of a topical eye anesthetic (0.4% oxybuprocaine hydrochloride eye drops, Novesin, Novartis, Switzerland) 1–2 min before. The injection of CBD, K1, K2, or olive oil was performed by oral gavage 1 h before each session of corneal stimulation.

Particularly, the stimulation (fixed current intensity of 44 mA, pulse width of 0.2 ms, duration of 3 s, frequency of 6-Hz) was delivered via corneal electrodes connected to a stimulator (ECT Unit 5780; Ugo Basile, Comerio, Italy).

### 4.10. Behavioral and ECoG Analysis

Seizures were defined as ECoG segments with a minimum duration of 5 s, continuous synchronous high-frequency activity, and an amplitude of at least twice the previous baseline. Notably, ECoG traces were digitally filtered offline (band-pass: high 50 Hz, low 1 Hz) and manually analyzed, using LabChart 8 PRO software (AD Instruments), by expert raters. The LabChart 8 PRO software was also used to observe power attenuation during the seizures induced by the last session of 6-Hz corneal stimulation. Particularly, the power attenuation gave a spectrum with a wide range of powers, and was expressed in logarithmic unit decibels (dB), relative to the reference power (attenuation = 10 log (power/reference power)).

Seizures were further analyzed using EDFbrowser (1st order Butterworth high-pass filter: 1 Hz; powerline interference removal: 50 Hz) [42] to understand whether significant changes in the cortical power band spectrum occurred during the induced seizures [35,36]. The power spectral density of the corresponding signal was shown as (µV)^2^/FFT-resolution. Moreover, a relative indication of the distribution of power over the frequency regions, ranging from 1 to 100 Hz, expressed in percentage (%) and recorded from the frontal electrodes, was determined. Especially, power band spectrum analysis was expressed in percentage, and included delta (δ, 0–4 Hz), theta (θ, 4–8 Hz), alpha (α, 8–12 Hz), beta (β, 12–24 Hz) and gamma (γ, 24–100 Hz) frequencies in 10-s epochs on a continuous ECoG.

Seizure severity was ranked according to the following scores: (i) stunned posture and eye blinking; (ii) head nodding, Straub tail and chewing; (iii) unilateral or alternating forelimb clonus; (iv) generalized tonic–clonic convulsions without loss of posture and rearing; (v) generalized tonic–clonic convulsions with loss of posture. Seizure severity and duration were first recorded through direct observation, and then reanalyzed on video recordings by an investigator unaware of the stimulation session. The time to recover from seizures was calculated from the end of the ECoG seizure to the reappearance of a normal exploratory behavior.

### 4.11. Immunohistochemistry

One hour after the last 6-Hz test, mice were deeply anesthetized with isoflurane for about 10 s and perfused transcardially with phosphate buffered saline (PBS, pH 7.4), followed by Zamboni’s fixative (pH 6.9). Brains were removed and postfixed at 4 °C in the same fixative for 24 h [18,19]. Tissues were transferred to 15% and 30% sucrose solutions, and then horizontal sections (50 μm thickness) were cut using a freezing-sliding microtome (Leica SM2000 R; Leica, Nussloch, Germany).

Sections were washed three times in PBS, treated with 3% H_2_O_2_ in PBS (10 min), and blocked for 1 h in PBS containing 5% normal goat serum and 0.1% Triton X-100. The sections were subsequently incubated overnight at 4 °C with rabbit polyclonal anti-FosB/ΔFosB (H-75, sc-7203, Santa Cruz Biotechnology, CA, USA; 1:250). The next day, the sections were incubated for 1 h with a biotinylated anti-rabbit secondary antibody (Vector Laboratories, Burlingame, CA, USA; 1:200). After washing, the brain sections were incubated for 1 h with the avidin-biotin-peroxidase complex (Elite ABC Kit; Vector Laboratories). The immunostaining was developed in 0.05% 3,3-diaminobenzidine tetrahydrochloride for 5 min (Sigma-Aldrich, Milan, Italy) by adding 0.03% H_2_O_2_.

### 4.12. Image Analysis

Immunostained sections from −8.04 mm to −5.04 mm Bregma level were evaluated with a Nikon Eclipse CiL (Nikon Instruments) at 20×, and for each area of interest (cornu ammonis 1 stratum pyramidalis—CA1 Py; Subiculum—Sub) images were digitally captured by a Nikon DS-Fi3 digital camera. The cellular density (n/mm^2^) of FosB/ΔFosB immunoreactive cells was quantified and analyzed using the ImageJ and NIS-Elements software. Particularly, FosB/ΔFosB-positive cells were counted by using ImageJ, whereas the region of interest (ROI) was manually traced in all analyzed sections by using NIS-Elements software.

### 4.13. Statistics

Fisher’s exact test was used to compare the number of animals experiencing convulsive seizures, with and without loss of posture, in the various treatment groups. The duration of the ictal ECoG recordings, and the time needed to recover from seizures, were analyzed by two-way (treatment × time) repeated measures ANOVA, followed by the Duncan’s method for post-hoc comparisons. Furthermore, the power band spectrum, expressed in percentage, was analyzed by two-way ANOVA, followed by Duncan’s method. Then, FosB/ΔFosB positivity values were compared using one-way ANOVA, followed by Duncan’s method. Statistical analyses were carried out using Sigmaplot 11 (Systat Software, San Jose, CA, USA). Data were represented as means, with standard error of the mean (SEM).

## 5. Conclusions

The present work was aimed at the preparation and chemical characterization of hemp oils, and at the evaluation of their in vivo antiepileptic effects in the 6-Hz corneal stimulation model. Olive oil formulations of medical *C. sativa*, currently used in patients, and oral administration, represent an easy route for animal testing as well.

The extraction procedure used in this work allowed us to obtain standardized hemp oils, as regards their cannabinoid content. The novelty of the present study is represented by the complete qualitative and quantitative characterization of terpenes in the extracts, with particular interest in the K2 hemp oil, which was prepared preserving volatile compounds.

The results obtained on the 6-Hz corneal stimulation model suggested a role of terpenic components in enhancing the antiepileptic activity of cannabinoids in K2 oil, compared to K1 and pure CBD, even if other minor compounds in the extracts can contribute to the overall activity.

Future work should be focused on the assessment of the bioactivity of K2 oil, as well as its main compounds, such as CBD and β-caryophyllene, on additional in vivo models of drug-resistant seizures, including Dravet and Lennox–Gastaut syndromes, although the high number of potentially active compounds in hemp extracts could complicate further studies on the mechanism of their antiseizure activity. The possible involvement of CB_2_ receptors in the observed antiseizure effect should also be evaluated.

## Figures and Tables

**Table 1:** Chemical formula, MS, and MS/MS data of the compounds identified in hemp oils both in the positive and negative ion mode by UHPLC-HRMS.

Compound	Formula	MS (*m*/*z*)	MS/MS (*m*/*z*)
Positive ion mode			
Cannflavin B	C_21_H_20_O_6_	369.1333	315.2315
Dehydrocannabifuran-C5 (DCBF)	C_21_H_24_O_2_	309.1775	239.8728
Epoxycannabigerol	C_21_H_32_O_3_	333.2424	315.2319
Cannabinodiol (CBND)	C_21_H_26_O_2_	311.2006	293.1898
Cannabidivarin (CBDV)	C_19_H_26_O_2_	287.0060	277.2160
Hydroxycannabidiol (OH-CBD)	C_21_H_30_O_3_	331.2268	313.2160
Cannabidiol-C4 (CBD-C4)	C_20_H_28_O_2_	301.2153	245.1530
Cannflavin A	C_26_H_28_O_6_	437.1959	359.2211
Cannabigerol (CBG)	C_21_H_32_O_2_	317.2475	245.1547
Cannabidiol (CBD)	C_21_H_30_O_2_	315.2319	245.1547
Cannabidiol monomethyl ether-C5 (CBDM)	C_22_H_32_O_2_	329.2474	313.1800
Bisnor-cannabielsoin-C1 (CBEO)	C_17_H_22_O_3_	275.1635	207.1013
Cannabinol (CBN)	C_21_H_26_O_2_	311.2006	293.1898
Δ^9^-Tetrahydrocannabinol (∆^9^-THC)	C_21_H_30_O_2_	315.2321	245.1547
Cannabichromene (CBC)	C_21_H_30_O_2_	315.2321	245.1547
Negative ion mode			
Hydroxycannabidiolic acid (OH-CBDA)	C_22_H_30_O_5_	373.2028	345.2076
Cannabidiolic acid-C4 (CBDA-C4)	C_21_H_28_O_4_	343.1932	325.1845
Cannabidiolic acid (CBDA)	C_22_H_30_O_4_	357.2021	313.2176
Cannabigerolic acid (CBGA)	C_22_H_32_O_4_	359.2228	331.2285
Δ^9^-Tetrahydrocannabinolic acid (Δ^9^-THCA)	C_22_H_30_O_4_	357.2021	313.2177
Cannabichromenic acid (CBCA)	C_22_H_30_O_4_	357.2021	313.2177

**Table 2:** Quantification of cannabinoids in hemp oils using HPLC-UV. Data expressed as mg/mL ± SD.

Peak Number	*t*_R_ (min)	Compound	Hemp Oil 1 (K1)	Hemp Oil 2 (K2)
1	11.6	CBDA	0.34 ± 0.01	<LOQ
2	13.4	CBGA	<LOQ	<LOQ
3	14.9	CBG	0.18 ± 0.03	0.20 ± 0.10
4	15.4	CBD	4.78 ± 0.23	4.24 ± 0.05
5	19.8	CBN	0.14 ± 0.03	0.14 ± 0.01
6	21.6	∆^9^-THC	0.42 ± 0.02	0.29 ± 0.01
7	23.6	∆^9^-THCA	<LOQ	<LOQ

LOQ = limit of quantification.

**Table 3:** Retention time, molecular formula, and molecular weight of the compounds identified in hemp oils by GC-MS.

Peak Number	*t*_R_ (min)	Compound	Formula	MW
1	8.6	α-Pinene	C_10_H_16_	136.23
2	9.3	Camphene	C_10_H_16_	136.23
3	10.7	β-Pinene	C_10_H_16_	136.23
4	11.5	β-Myrcene	C_10_H_16_	136.23
5	12.2	α-Phellandrene	C_10_H_16_	136.23
6	12.4	∆^3^-Carene	C_10_H_16_	136.23
7	12.5	Limonene	C_10_H_16_	136.23
8	17.0	α-Terpinolene	C_10_H_16_	136.23
9	17.9	Linalool	C_10_H_18_O	154.25
10	23.3	p-Cymen-8-ol	C_10_H_14_O	150.22
11	33.4	Ylangene	C_15_H_2_	204.35
12	34.9	β-Caryophyllene	C_15_H_24_	204.35
13	35.5	α-Bergamotene	C_15_H_24_	204.35
14	36.0	α-Humulene	C_15_H_24_	204.35
15	38.6	*trans*-Nerolidol	C_15_H_26_O	222.37
16	39.8	Caryophyllene oxide	C_15_H_24_O	220.35

**Table 4:** Quantification of terpenes in hemp oils using GC-MS. Data expressed as µg/mL ± SD.

Compound	Hemp Oil 1 (K1)	Hemp Oil 2 (K2)
α-Pinene	3.48 ± 0.18	11.65 ± 0.98
β-Pinene	<LOQ	<LOQ
β-Myrcene	<LOQ	7.29 ± 0.19
Limonene	<LOQ	8.99 ± 0.46
Linalool	<LOD	5.29 ± 0.24
β-Caryophyllene	<LOQ	26.61 ± 3.00
α-Humulene	<LOQ	9.74 ± 1.08
*trans*-Nerolidol	<LOQ	4.15 ± 0.77
Caryophyllene oxide	<LOQ	8.25 ± 1.55

LOQ = limit of quantification. LOD = limit of detection.

## Data Availability

The data presented in this study are available on request from the corresponding authors.
